# Data set for mass spectrometric analysis of recombinant human serum albumin from various expression systems

**DOI:** 10.1016/j.dib.2015.07.024

**Published:** 2015-08-01

**Authors:** Daryl G.S. Smith, Grant E. Frahm, Anita Kane, Barry Lorbetskie, Michel Girard, Michael J.W. Johnston, Terry D. Cyr

**Affiliations:** Centre for Biologics Evaluation, Biologics and Genetic Therapies Directoriate, Health Canada, Ottawa, ON, Canada K1A0K9

**Keywords:** Albumin, Recombinant proteins, Glycation, Mass spectrometry, *Oryza sativa*

## Abstract

Human serum albumin (HSA) is a versatile and important protein for the pharmaceutical industry (Fanali et al., Mol. Aspects Med. 33(3) (2012) 209–290). Due to the potential transmission of pathogens from plasma sourced albumin, numerous expression systems have been developed to produce recombinant HSA (rHSA) (Chen et al., Biochim. Biophys. Acta (BBA)—Gen. Subj. 1830(12) (2013) 5515–5525; Kobayashi, Biologicals 34(1) (2006) 55–59). Based on our previous study showing increased glycation of rHSA expressed in Asian rice (Frahm et al., J. Phys. Chem. B 116(15) (2012) 4661–4670), both supplier-to-supplier and lot-to-lot variability of rHSAs from a number of expression systems were evaluated using reversed phase liquid chromatography linked with MS and MS/MS analyses. The data are associated with the research article ‘Determination of Supplier-to-Supplier and Lot-to-Lot Variability in Glycation of Recombinant Human Serum Albumin Expressed in *Oryza sativa*’ where further analysis of rHSA samples with additional biophysical methods can be found (Frahm et al., PLoS ONE 10(9) (2014) e109893). We determined that all rHSA samples expressed in rice showed elevated levels of arginine and lysine hexose glycation compared to rHSA expressed in yeast, suggesting that the extensive glycation of the recombinant proteins is a by-product of either the expression system or purification process and not a random occurrence.

**Specifications table**

**Table:** 

Subject area	Biology
More specific subject area	Recombinant proteins
Type of data	Raw Data, Peak Lists, Search Results and Exported Search Results
How data was acquired	Liquid chromatography–mass spectrometry (LC–MS) analysis—waters nanoAcquity UPLC and waters synapt HDMS system operating in data dependant acquisition (DDA) mode.
Data format	Waters raw data (.raw), Mascot generic file peak lists (.mgf), Mascot search results (.dat) and exported search results (.mzid)
Experimental factors	Samples were reduced and then alkylated with iodoacetamide followed by digestion with trypsin and chymotrypsin
Experimental features	Commercially available recombinant human serum albumin samples were digested and analyzed by LC–MS/MS
Data source location	Ottawa, Ontario, Canada
Data accessibility	Data have been deposited to the ProteomeXchange Consortium via the PRIDE partner repository (PXD001248 and DOI 10.6019/PXD001248).

**1. Value of the data** [describe in 3–5 bulleted points why this data is of value to the scientific community]

• Represents a robust method for profiling variation in glycation of commercial products
• Demonstrates the utility of multi-enzyme approach for extensive protein sequence coverage allowing detailed analysis/comparison of non-complex proteomic samples
• Provides a model data set for qualitative and quantitative proteomics studies
• Covers the entire data generation/analysis process, from raw data, to processed peak lists, to search results, to exported result files.

## 2 Experimental design, materials and methods

As described in ‘Determination of Supplier-to-Supplier and Lot-to-Lot Variability in Glycation of Recombinant Human Serum Albumin Expressed in *Oryza sativa*’ [1,2,3,5].

## 3 Materials

Chemicals, essentially FA-free pHSA (A3872 Lot#090M7001V, ≥99% purity), recombinant human serum albumin expressed in Saccharomyces cerevisiae (ScrHSA, Lot# SLBD2407, ≥99% purity, Albucult), *O. sativa* [OsrHSA, Lot# SLBC7527V (OsrHSA-sig-C), Lot# SLBG7405V (OsrHSA-sig-G), Lot# SLBH9636V (OsrHSA-sig-H) and Lot# SLBJ1196V (OsrHSA-sig-J) 100% purity, Cellastim] and Pichia pastoris (PprHSA, Lot# 080M1580V, ≥99% purity, Albagen) were sourced from Sigma-Aldrich (St. Louis, MO, USA). OsrHSA was also obtained from eEnzyme LLC (Gaithersburg, MD, USA) (OsrHSA-phy) (Lot# 20130110, >99% purity, Phyto-HSA), ScienCell Research Laboratories (Carlsbad, CA, USA) (OsrHSA-sci) (Lot# BJABAA42, ≥99% purity, Oryzogen) and amsbio LLC (Cambridge, MA, USA) (OsrHSA-ams) (Lot #20101008, >95% purity, ecoHSA). Recombumin (Lot# PDP100106) was donated by Novozymes Biopharma (Cambridge, MA, USA). Amicon Ultra 0.5 ml 3000 Da molecular weight cut-off (MWCO) centrifugal filter devices were purchased from Millipore (Billerica, MA, USA). Trypsin and chymotrypsin were Promega sequencing grade purchased from Fisher Scientific Canada (Ottawa, ON, Canada). Vivacon 500 10 kDa molecular weight cut-off filters were from Sartorius Stadium Biotech North America (Bohemia, NY, USA).

## 4 Albumin sample preparation

Albumin samples were prepared as described previously [4]. Briefly, samples were buffer exchanged into 5 mM sodium phosphate (pH 7.4), with Amicon Ultra 0.5 ml 3000 Da MWCO centrifugal filter devices after pre-rinsing the filters with buffer. Protein concentrations were measured using a BCA assay kit (Sigma-Aldrich, St. Louis, MO, USA). Protein integrity after buffer exchange was assessed with 1-D SDS−PAGE using SYPRO Ruby protein stain (Molecular Probes, Eugene, OR, USA) and a Bio-Rad Molecular Imager Gel Doc XR+ system with Quantity One 1-D analysis software according to the manufacturer’s instructions (Bio-Rad, Mississauga, ON, Canada).

## 5 Liquid chromatography–mass spectrometry (LC–MS) analysis—Sample preparation

For each of the rHSA samples, approximately 10 µg were diluted in 50 mM ammonium bicarbonate to a total volume of 200 µl. To each solution 10 µl of 250 mM dithiothreitol (DTT) in 50 mM NH_4_HCO_3_ were added followed by incubation for 1 h at 60 °C. Next, 20 µl of 250 mM iodoacetamide in 50 mM NH_4_HCO_3_ were added and samples were incubated at room temperature in the dark for 30 min. The alkylation reactions were quenched by the addition of an additional 20 µl of 250 mM DTT and the samples were split into 2×125 µl aliquots and transferred onto 10 kDa MWCO spin filters. Samples were centrifuged for 20 min at 14,000×*g* then washed with 2×200 µl of 50 mM NH_4_HCO_3_ in the same manner.

Trypsin and chymotrypsin solutions were prepared by adding 1000 µl of 50 mM NH_4_HCO_3_ to 20 µg and 25 µg lyophilized protein, respectively. One 125 µl aliquot from each sample was digested with trypsin and the other with chymotrypsin. Digestion was carried out by spinning 100 µl of trypsin solution through the filters at 10,000×*g* over 20 min, then spinning 100 µl of 50 mM NH_4_HCO_3_ through the filter under the same conditions. The flow through from the digestion steps was collected in clean tubes and evaporated to dryness in a vacuum centrifuge then re-suspended in 40 µl of injection solvent (3% acetonitrile, 0.2% formic acid and 0.05% TFA in water) prior to LC–MS/MS analysis.

## 6 LC–MS analysis—Sample analysis

For each sample, triplicate 2 µl aliquots were analyzed by loading onto a Waters Symmetry C18 trap column (180 µm×20 mm with 5 µm beads) and desalting with 0.1% formic acid in water (solvent A) for 3 min at 5.0 µl/min before separating on a Waters nanoAcquity UPLC BEH130 C18 reverse phase analytical column (100 µm×100 mm with 1.7 µm beads). Chromatographic separation was achieved at a flow rate of 0.500 µl/min over 70 min in six linear steps as follows (solvent B was 0.1% formic acid in acetonitrile): Initial—3% B, 2 min—10% B, 40 min—30% B, 50 min—95% B, 55 min—95% B, 56 min—3% B, final—3% B. The eluting peptides were analyzed by MS and MS/MS using a Waters Synapt HDMS system operating in data directed acquisition (DDA) mode. MS survey scans were 1 s in duration and MS/MS data were collected on the four most abundant peaks until either the total ion count exceeded 3000 or until 3 s elapsed. Within each analysis, redundant analyses were limited by excluding selected peaks ±1.15 mass-to-charge (*m*/*z*) for 60 s. Between triplicate analyses, previously selected peaks were prevented from being reanalyzed by using *m*/*z* (±1.15) and retention time (±60 s) as exclusion criteria. Peaks from singly-charged peptides were also excluded from selection for MS/MS analysis. The instrument was calibrated prior to sample analysis using the fragmentation products of [Glu1]-Fibrinopeptide B. Calibration accuracy was maintained throughout the analyses using a nano-lock spray of 100 fmol/µl [Glu1]-Fibrinopeptide B, which was sampled for 1 s once every 30 s. The lock mass correction was applied to the data during processing.

## 7 LC–MS analysis—Qualitative data processing

Data were qualitatively analyzed using the Mascot software package, available from Matrix Science Ltd. (Boston, MA, U.S.A.). The raw data were processed using Mascot Distiller (version 2.4.2) to create Mascot Generic Files (MGFs) and database searches were performed using Mascot (version 2.4), against the human protein entries in the 2013_01 UniProtKB/Swiss-Prot database. MGF files from the triplicate analyses of both the trypsin and chymotrypsin digests were combined and submitted as a single search for each sample. Peptide and MS/MS mass tolerances were 100 ppm and 0.1 Da, respectively, and semi-tryptic and semi-chymotryptic peptides, from 2+ to 5+ charge state and having up to three missed cleavages, were considered. Carbamidomethylation of cysteine was specified as a fixed modification and oxidation of methionine and hexose addition on lysine and arginine were considered as variable modifications. The mass spectrometry proteomics data have been deposited to the ProteomeXchange Consortium via the PRIDE partner repository with the dataset identifier PXD001248 and DOI 10.6019/PXD001248.

## References

[1] Fanali G, di MA, Trezza V, Marino M, Fasano M, Ascenzi P. (2012). Human serum albumin: from bench to bedside. Mol. Aspects Med..

[2] Chen Z, He Y, Shi B, Yang D. (2013). Human serum albumin from recombinant DNA technology: challenges and strategies. Biochim. Biophys. Acta (BBA)—Gen. Subj..

[3] Kobayashi K. (2006). Summary of recombinant human serum albumin development. Biologicals.

[4] Frahm GE, Cyr TD, Smith DG, Walrond LD, Johnston MJ. (2012). Investigation of the differences in thermal stability of two recombinant human serum albumins with 1,2-dipalmitoyl-sn-glycero-3-phosphocholine liposomes by UV circular dichroism spectropolarimetry. J. Phys. Chem. B.

[5] Frahm GE, Smith DG, Kane A, Lorbetskie B, Cyr TD, Girard M (2014). Determination of supplier-to-supplier and lot-to-lot variability in glycation of recombinant human serum albumin expressed in *Oryza sativa*. PLoS ONE.

